# The Interleukin-17 Receptor B Subunit Is Essential for the Th2 Response to *Helicobacter pylori*, but Not for Control of Bacterial Burden

**DOI:** 10.1371/journal.pone.0060363

**Published:** 2013-03-22

**Authors:** Dennis J. Horvath, Jana N. Radin, Sung Hoon Cho, M. Kay Washington, Holly M. Scott Algood

**Affiliations:** 1 Veterans Affairs Tennessee Valley Healthcare System, Nashville, Tennessee, United States of America; 2 Departments of Medicine, Vanderbilt University School of Medicine, Nashville, Tennessee, United States of America; 3 Pathology, Microbiology, and Immunology, Vanderbilt University School of Medicine, Nashville, Tennessee, United States of America; Veterans Affairs Medical Center (111D), United States of America

## Abstract

*Helicobacter pylori* infection leads to an inflammatory response in 100% of infected individuals. The inflammatory cells which are recruited to the gastric mucosa during infection produce several pro- and anti-inflammatory cytokines including several cytokines in the interleukin-17 family. The anti-inflammatory cytokine, interleukin 25 (IL-25, also known as IL-17E), signals through a receptor, which is a heterotrimeric receptor comprised of two IL-17 receptor A subunits and an IL-17 receptor B subunit. Previous studies in our laboratory demonstrated that IL-17RA is required to control infection with *Helicobacter pylori* in the mouse model. Moreover, the absence of IL-17 receptor A leads to a significant B cell infiltrate and a remarkable increase in lymphoid follicle formation in response to infection compared to infection in wild-type mice. We hypothesized that IL-25, which requires both IL-17 receptor A and IL-17 receptor B for signaling, may play a role in control of inflammation in the mouse model of *Helicobacter pylori* infection. IL-17 receptor B deficient mice, IL-17 receptor A deficient mice and wild-type mice were infected with *Helicobacter pylori* (strains SS1 and PMSS1). At several time points *H. pylori*- infected mice were sacrificed to investigate their ability to control infection and inflammation. Moreover, the effects of IL-17 receptor B deficiency on T helper cytokine expression and *H. pylori*- specific serum antibody responses were measured. IL-17 receptor B−/− mice (unlike IL-17 receptor A−/− mice) exhibited similar or modest changes in gastric colonization, inflammation, and Th1 and Th17 helper cytokine responses to wild-type mice infected with *Helicobacter pylori*. However, *H. pylori*-infected IL-17 receptor B−/− mice have reduced expression of IL-4 and lower serum IgG1 and IgG2a levels compared to infected IL-17 receptor A−/− and wild-type mice. These data indicate that signaling through the IL-17 receptor B subunit is not necessary for control of *Helicobacter pylori* in our model.

## Introduction

*Helicobacter pylori* is a gram-negative microaerophilic bacterium, which colonizes the gastric mucosa in about 50% of the world's population [1], [2]. While there is a robust immune response to the bacterium, the response is ineffective and unless patients are treated with antibiotics they will harbor *H. pylori* for their lifetime. A majority of colonized persons will not develop symptoms, but all colonized persons are believed to develop an inflammatory response, termed gastritis [3]. In some individuals, chronic gastritis is the first step in a pathway that leads to more adverse outcomes including gastric adenocarcinoma, MALT lymphoma, or duodenal ulcers [4].

Gastritis is dependent on a robust T cell-mediated response, which involves a mixed T lymphocytes response [reviewed in [5]]. These lymphocytes produce a number of T cell-derived cytokines, including IL-4, and several proinflammatory cytokines IFNγ, IL-17A, and IL-17F, which then recruit and activate effector cells such as neutrophils, macrophages and B lymphocytes. The proinflammatory response is regulated in most individuals by T regulatory cells and the production of anti-inflammatory cytokines such as IL-10 ([6], [7], [8], [9], [10]–[12] and reviewed in [13]).

Recent interest in identifying which factors contribute to control of colonization and inflammation led us to investigate the contribution of some of the IL-17 family members. IL-17A and IL-17F are produced during *H. pylori* infection [9], [14]–[18] and Th17 cells contribute to control of infection and to chronic inflammation in many models of *H. pylori* disease (reviewed in [19]). Functional receptors for IL-17 family cytokines are thought to consist of homodimers or heterodimers [20], [21]. For example, the heterodimer of IL-17 receptor A subunit and IL-17 receptor C subunit is a receptor for homodimers and heterodimers of IL-17A and IL-17F [22], whereas the heterodimer consisting of IL-17RA and IL-17 receptor B subunit serves as a receptor for IL-17E [23]. Both IL-17B and IL-17E bind to IL-17RB [24].

We began our investigations utilizing the IL-17RA-deficient mice, which are deficient for signaling for IL-17A, IL-17C [25], IL-17E [26], IL-17F and IL-17A/F. In our previously published work [17], we demonstrate that *H. pylori-* infected IL-17RA−/− mice have a neutrophil recruitment defect and do not control *H. pylori* bacterial burden as well as wild-type (WT) mice. Most notably and unique to the *H. pylori* model of infection, we found that IL-17RA signaling was necessary to limit B cell infiltration to the gastric mucosa. In the absence of IL-17RA, not only was there an increase in total numbers of B cells in the stomach, but an increase in the number of lymphoid follicles with germinal centers. This led us to question whether IL-17RA may have a role in limiting inflammation.

Our findings in the IL-17RA−/− mice cannot be recapitulated in IL-17A−/− or with neutralization of IL-17A [17], [27]–[30]. IL-17 deficiencies in *H. pylori* infection models lead to reduced neutrophilic infiltration, but there has been some inconsistency (likely due to inconsistent time courses in the models) as to whether IL-17A and IL-17F are required to control *H. pylori* colonization [17], [27]–[30]. While there has not been consistent results concerning colonization and inflammation in studies using IL-17A−/− mice or in IL-17 neutralization studies, no study has demonstrated the remarkable increase in B cell infiltration and lymphoid follicle formation, which we demonstrated in the *H. pylori-* infected IL-17RA−/− mice [17]. This led us to several hypotheses. One hypothesis was that IL-17RB and IL-17E may contribute to control of gastric inflammation during *H. pylori* infection since IL-17RA is also necessary for signaling for IL-17E. The IL-17E receptor is a heterodimer of IL-17RA and IL-17RB. IL-17E, also known as IL-25, is the most distant member of the IL-17 family (compared to IL-17A), sharing only 17% homology [26]. IL-17E has primarily believed to amplify T helper 2 responses [31], [32], but can also suppress Th1 and Th17 responses. IL-17E has been described to induce IL-8 homologs and Th2 cytokines in the mouse.

In this study, utilizing an *H. pylori* mouse model, *H. pylori-*infected IL-17RB-deficient mice show a similar pattern of cellular infiltration as *H. pylori-*infected WT mice, similar levels of inflammation and bacterial burden. Th2 responses were mildly suppressed in the absence of IL-17RB signaling during *H. pylori* infection compared to WT responses. In conclusion, we demonstrate that while IL-17RB is necessary for an *H. pylori-* specific IgG1 response, IL-17E signaling is not essential for control of *H. pylori* colonization and inflammation.

## Materials and Methods

### Animals

Permission to use male and female IL-17RA−/− and IL-17RB−/− mice for the establishment of a breeding colony was obtained from Amgen (Seattle, WA). These generation of these mice was previously described, IL-17RA−/− [33] and IL-17RB−/− [23]. Amgen's IL-17RA−/− and IL-17RB−/− mouse breeding colony is maintained at Taconic Farms. *Helicobacter*-free IL-17RB−/−, IL-17RA−/− and WT mice (all C57BL/6 background; Taconic Farms, Germantown, NY), 8–10 weeks old, were used in all experiments. Fecal samples were collected and sent to the Research Animal Diagnostic and Investigation Laboratory (RADIL; University of Missouri) for testing of intestinal *Helicobacter* by PCR. All breeding pairs tested negative. Mice were confirmed positive for segmented filamentous bacteria by standard Sybr Green PCR assay on the Applied Biosystems StepOne Plus Instrument (primers and methods were previously described [34]). Feces from sentinel mice housed in the same room were routinely tested by PCR for intestinal *Helicobacter*, pinworms, mouse parvovirus and several other murine pathogens, and consistently tested negative for each of these infections.

### Ethics statement

The Vanderbilt University Institutional Animal Care and Use Committee and the Department of Veteran's Affairs committee approved all animal protocols used in this study (ACORP V/10/410).

### Culture of *H. pylori*

A mouse-passaged derivative of *H. pylori* strains SS1 or PMSS1 were used in all experiments [35], [36]. These strains were a gift from Dr. Timothy Cover (Vanderbilt University). Bacteria were grown on trypticase soy agar (TSA) plates containing 5% sheep blood. Alternatively, bacteria were grown in *Brucella* broth containing 5% heat-inactivated fetal bovine serum (FBS) and 10 μg/ml vancomycin. Cultures were grown at 37°C in either room air supplemented with 5% CO_2_, or under microaerobic conditions generated by a CampyPak Plus^*^ Hydrogen + CO_2_ with Integral Palladium Catalyst (BD).

### Infection of mice with *H. pylori* and harvest procedures

One day prior to infection of mice, *H. pylori* were inoculated into liquid medium and were cultured for 18 hours under microaerobic conditions, as described above. Mice were orogastrically inoculated with a suspension of 5×10^8^ CFU *H. pylori* (in 0.5 ml of *Brucella* broth) twice over 5 days. At animal sacrifice tissue and serum were collected for various analyses including flow cytometry, cytokine expression analysis, histology (inflammation scoring), and antibody responses. While serum was collected from all mice, the stomach was divided for analyses. Some analysis of stomach tissue requires more tissue. Therefore, all the analyses on the stomach cannot be performed on one piece of tissue. This explains why the numbers of animals for the antibody titers is higher than some of the other analyses.

### Processing of mouse stomachs

The stomach was removed from each mouse by excising between the esophagus and the duodenum. The forestomach (nonglandular portion) was removed from the glandular stomach and discarded. The glandular stomach was opened, rinsed gently in cold PBS, and cut into three longitudinal strips that were used for bacterial culture, RNA analysis, and histology. For culturing of *H. pylori* from the stomach, gastric tissue was placed into *Brucella* broth-10% FBS for immediate processing. Gastric tissue was stored in RNALater solution for subsequent RNA isolation. A longitudinal strip from the greater curvature of the stomach was excised and placed in 10% normal buffered formalin for 24 hours, embedded in paraffin and processed routinely for hematoxylin and eosin (H&E) staining. Indices of inflammation and injury were scored by a single pathologist (KW) who was blinded to the identity of the mice. Acute and chronic inflammation in the gastric antrum and corpus were graded on a 0–3 scale. Acute inflammation was graded based on density of neutrophils and chronic inflammation was graded based on the density of lamina propria mononuclear cell infiltration independent of lymphoid follicles. Total inflammation was calculated as a sum of acute and chronic inflammation in the corpus and the antrum allowing for quantification of total inflammation on a scale of 0–12.

### Culture of *H. pylori* from mouse stomach

Gastric tissue was homogenized using a tissue tearor Biospec (BioSpec Products, Inc. Bartlesville, OK). Serial dilutions of the homogenate were plated on trypticase soy agar plates containing 5% sheep blood, 10 μg/ml nalidixic acid, 100 μg/ml vancomycin, 2 μg/ml amphotericin, and 200 μg/ml bacitracin. After 5 to 7 days of culture under microaerobic conditions, *H. pylori* colonies were counted.

### RNA extraction and real-time rtPCR

RNA was isolated from the stomach using the TRIZOL isolation protocol (Invitrogen, Carlsbad, CA) with slight modifications as previously described [37]. RNA was reverse transcribed using the High Capacity cDNA Reverse Transcription Kit (Applied Biosystems, Foster City, CA). For real time rtPCR, we used the relative gene expression method [38]. Glyceraldehyde 3-phosphate dehydrogenase (GAPDH) served as the normalizer, and tissue from uninfected WT mouse stomachs served as the calibrator sample. All real time rtPCR was performed using an Applied Biosystems StepOne Plus real time PCR instrument. Levels of cytokine expression are indicated as “relative units”, based on comparison of tissue from *H. pylori*-infected mice with tissue from uninfected mice (calibrator tissue) [38]. Primer and probe sets were purchased as Taqman Gene Expression Assays from Applied Biosystems (as pre-designed assays the annealing temperatures and amplicon length are available on their website). Primer and probe sets were purchased as Taqman Gene Expression Assays from Applied Biosystems [IL-4 (Mm00445260_m1), IL-17a (Mm00439619_m1), IL-17E/IL-25 (Mm00499822_m1), GAPDH (Mm99999915_g1), IFNγ (Mm99999071_m1)].

### Flow cytometric analysis

To analyze gastric cellular infiltrates, whole mouse stomachs were harvested and processed using Miltenyi's Gentle Dissociator (Miltenyi Biotec, Boston, MA). In brief, the stomach was cut into 5 mm pieces and then transferred to a C-tube (Miltenyi Biotec) in 5 mL RPMI/10% FBS. The preset Miltenyi Biotec program m_imptumor 02 was run once and then the tissue was digested for 30 minutes at 37°C in a solution containing (0.32 mg/mL Dispase and 0.30 mg/mL Collagenase D, Roche)while shaking in a CO_2_ incubator. After the 37°C incubation, 100 U/mL of DNase (Sigma D4527) was added to each tube and the Miltenyi Gentle Dissociator was run for a second and third time using the preset program m_imptumor 02. The tissue homogenate was passed through a 70 μm cell strainer (BD). Cells were harvested by centrifugation, washed, and then live cells were counted by using a hemocytometer and trypan blue exclusion staining. The samples were stained with 2 μg/ml anti-CD4 (clone H129.19), anti-CD8a (clone 53–6.7, and anti-CD3e (clone 1145-2C11), or 1.5 μg/ml anti-Gr1 (clone RB6-8C5), 2 μg/ml anti-CD11b (clone M1/70) and anti-B220 (RA3-6B2) (all antibodies were purchased from BD Bioscience, San Jose California) in a volume of 100 μl FACS buffer (PBS, pH 7.4, containing 0.1% sodium azide, 0.1% BSA, and 20% mouse serum). Cells were washed, resuspended in FACS buffer, and analyzed on a BD LSR II flow cytometer (BD Bioscience).

### ELISAs for *H. pylori*- specific antibodies

Isotype antibodies for *H. pylori* were quantitated by enzyme-linked immunosorbent assay (ELISA). Plates were coated with 100 µl *H. pylori* SS1 lysates (10 μg/ml) overnight at 4°C, washed three times with wash buffer (0.05% Tween 20 in PBS) and blocked with blocking buffer (5% skim milk, 0.05% Tween 20 in PBS) for 1 h. Serum samples (100 µl/well) were added in duplicate, and incubated for 2 h at room temperature. Plates were washed five times with wash buffer followed by addition of detection Abs [goat anti-mouse IgG1-HRP, goat anti-mouse IgG2a-HRP, or goat anti-mouse IgA-HRP (Southern Biotechnology, Birmingham, AL)] for 1 h at room temperature. After washing five times with wash buffer, color was developed by addition of 100 µl 1-Step^TM^ Ultra TMB-ELISA (Thermo Scientific, Rockford, IL). The reaction was stopped by adding 2N H_2_SO_4_, and plates were read at 450 nm. Titers presented in the table are expressed as the reciprocal of the dilution of serum that yielded an optical density at 450 nm twice the background signal generated by sera from uninfected mice. If the optical density at 450 nm was not twice the level of the background signal then the antibody levels were considered below the limit of detection (BD, below detection).

### Statistical analysis

Four to ten mice per group per time point were used for all of the studies. To compare results obtained with different groups of mice, statistical analysis was performed using ANOVA followed by a t-test for multiple comparisons. For analyses of bacterial numbers, the data were normalized by log transformation prior to statistical analysis. For histology scores, the Mann-Whitney U-test was applied to compare results between IL-17RA−/−, WT mice and IL-17RA−/− mice. To test statistical power between the presence and absence of lymphoid follicles or the loss of parietal cells a Chi-squared analysis was performed. Statistical analyses were performed using GraphPad Prism Software.

## Results and Discussion

### IL-25/IL-17E expression increases in the stomach after *H. pylori* infection in the mouse model

**Figure 1:**
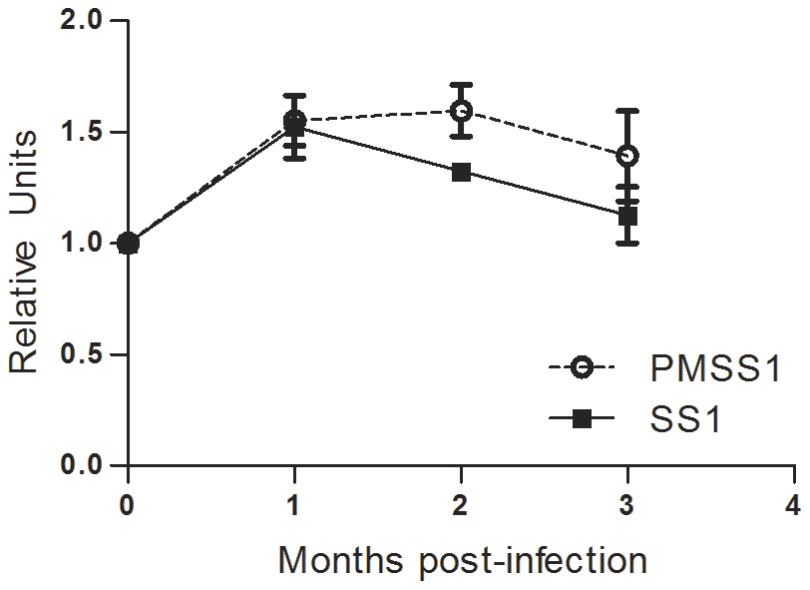
Expression of IL-17RB ligand, IL-17E (IL-25) following *H. pylori* infection in mice. Wild-type C57BL/6 mice were infected with *H. pylori* strain PM-SS1 or SS1 and expression of IL-17E was measured in the gastric tissue by real time rtPCR over 3 months. Relative units also known as normalized expression are a measure of the relative expression calibrated to expression in uninfected wild-type mice using GAPDH as the endogenous control. Each time point represents 5 mice per group.

To determine if IL-17E (IL-25) expression is influenced by *H. pylori* infection, we used real time RT-PCR to measure the expression of IL-17E in the stomach of *H. pylori* infected WT C57BL/6 mice, relative to uninfected stomach tissue. We find a consistent increase in the expression of IL-17E by 1 month post-infection and our chronic infection time point of 3 months post-infection (Figure 1). These analyses were performed with 2 strains of *H. pylori* (SS1 and PMSS1, [39]) that differ in the presence of a functional *cag* pathogenicity *island*, an important virulence determinant of *H. pylori* [40]. Similar gastric expression of IL-17E between these two different strains suggested that a functional *cag* pathogenicity island is not necessary for induction of IL-17E.

### IL-17RB−/− mice control *H. pylori* colonization

**Figure 2:**
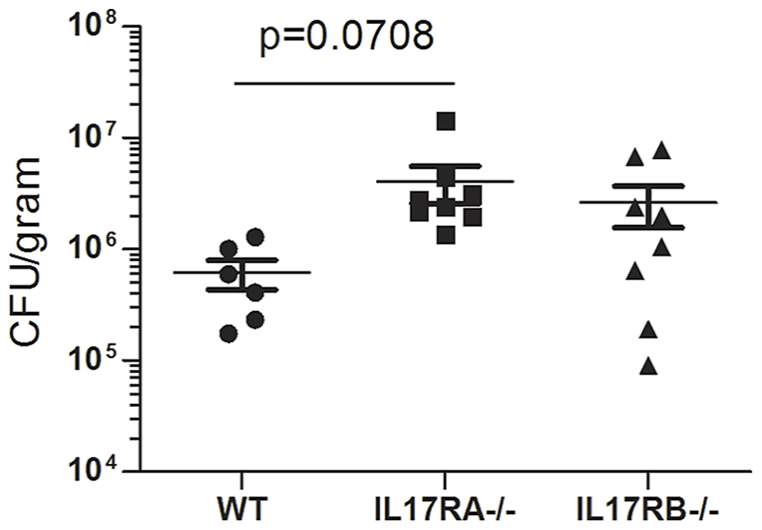
Colonization of IL-17RB deficient mice with *H. pylori*. IL-17RA−/− mice, IL-17RB−/− mice and WT mice were infected with *H. pylori* strain SS1. Levels of colonization were measured by plating serial dilutions of stomach homogenates. The number of colony forming units (CFU) was then calibrated to the weight of the tissue and CFU/gram is presented on the graphs for 3 months post-infection.

In our previously published studies, we described an essential role for IL-17RA signaling for control of bacterial burden [17]. Since IL-17RA is required for signaling of several cytokines, we investigated whether IL-17E could be contributing to the phenotype by using another model with an IL-17E signaling deficiency, the IL-17RB−/− mouse. IL-17RA−/−, IL-17RB−/− and WT C57BL/6 mice were orogastrically infected with 2 doses of *H. pylori* (either strain SS1 or PMSS1). At several time points, 1, 2, and 3 months post infection, mice were sacrificed for analyses. Our results indicate that, IL-17RBcontrol *H. pylori* colonization of the stomach better than IL-17RA−/−. While there is a modest increase in the bacterial burden in the IL-17RB−/− mice compared to WT mice, the difference is not statistically significant (Strain SS1, Figure 2; Strain PMSS1 data not shown).

### IL-17RB signaling influences gastric inflammation and cellular infiltrates

**Figure 3:**
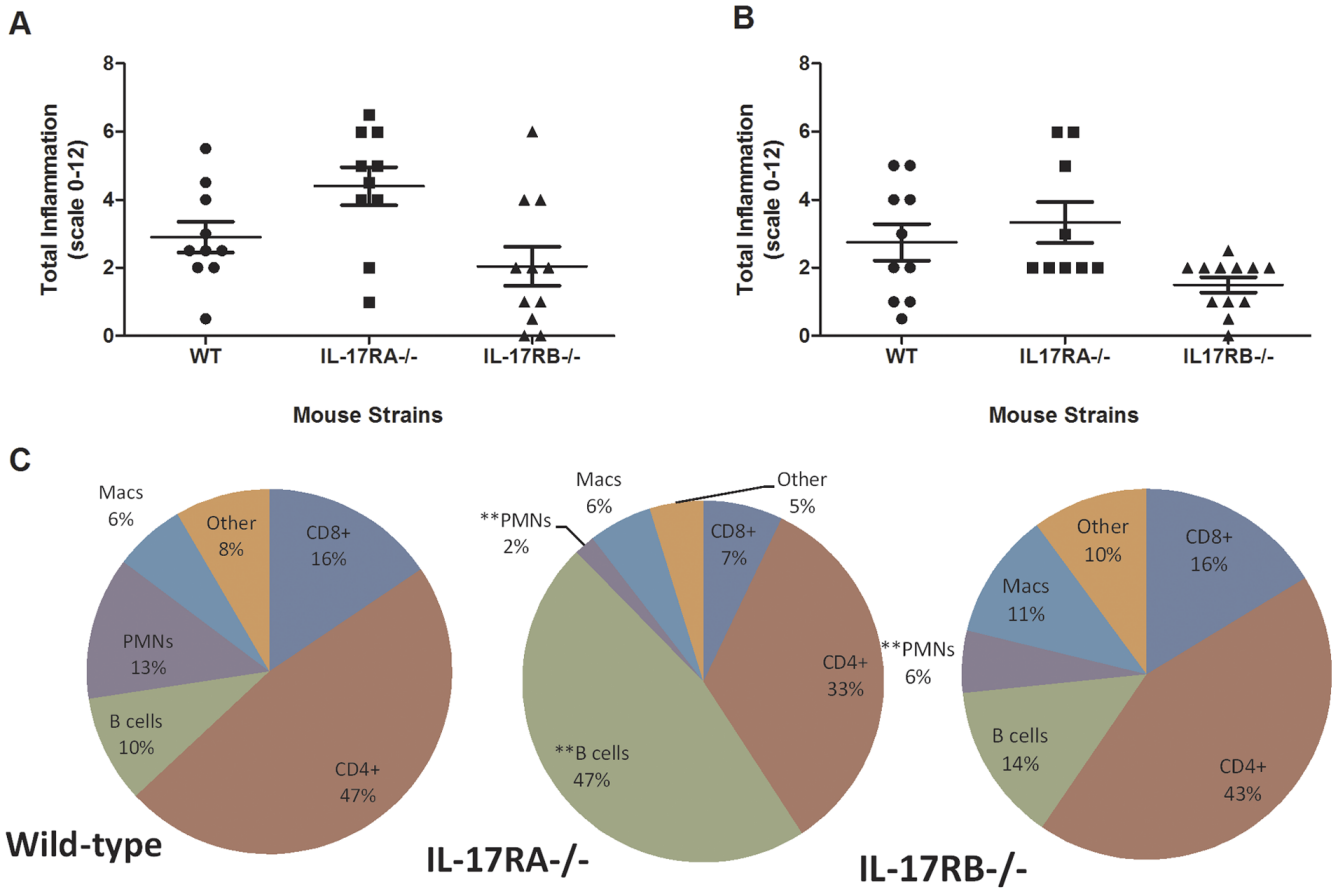
Inflammation is increased in *H. pylori*-infected IL-17RA−/− mice compared to *H. pylori*-infected IL-17RB−/− and wild-type mice. Levels of acute and chronic inflammation were scored on stomach tissue (in the corpus and antrum) at 2 months (A) and 3 months (B) post infection with strain SS1. Total inflammation was scored on a scale of 0–12. The inflammatory cells were immunophenotyped in the gastric mucosa using flow cytometry (C). The pie charts represent the percentage of specific types of immune cells in the lymphocyte/granulocyte gate. The percentage is an average of 4–5 mice per group. *p≤0.05, **p≤0.01, compared to *H. pylori*-infected wild type percentages.

IL-17RA signaling is required to control B cell infiltration into the stomach during *H. pylori* infection [17], since *H. pylori-* infected IL-17RA−/− mice have increased B cell infiltration, increased numbers of lymphoid follicles and stronger *H. pylori-*specific serum antibody responses compared to WT mice. But, these findings are not consistent with studies, which focused on the individual role of IL-17A [29], which signals through IL-17RA. To investigate whether IL-17E signaling was also contributing to the phenotype observed in the IL-17RA−/− mice, we assessed the levels of gastric inflammation in the *H. pylori-* infected IL-17RB−/− mice compared to both infected IL-17RA−/− mice and WT mice. Our results indicate that IL-17RB−/− mice have similar levels of gastric inflammation as WT mice at 2 months post-infection (Figure 3A, Table 1) At 3 months post infection the IL-17RB−/− have a lower level of inflammation than both the WT and the IL-17RA−/− mice (Table 1, Figure 3B). Moreover, the percentage of B cell in the *H. pylori-* infected IL-17RB−/− mice were similar to the percentage of B cells in the *H. pylori-* infected WT mice, but different from *H. pylori-* infected IL-17RA−/− mice, which as expected, exhibited a strong B cell response in the stomach (Figure 3C, p = 0.007). The only other significant difference in the percentage of cells, which migrated into the gastric mucosa in this study, was in the numbers of neutrophils. As expected the *H. pylori-* infected IL-17RA−/− mice had significantly reduced levels of neutrophils compared to *H. pylori-*infected WT mice (Figure 3C, p = 0.0003), and, while the difference was not as striking, there was also a significant reduction in the percentage of neutrophils in the *H. pylori* – infected IL-17RB−/− mice compared to the *H. pylori* -infected WT mice (Figure 3C, p = 0.0073) which supports previous findings that IL-17E may induce IL-8 homologs in the mouse. The differences in the other cell types between IL-17RB−/− and WT mice are not significant.

**Table 1:** Histological findings in *H. pylori-* infected mice (strain SS1).

Strain of mice	Lymphoid follicles (# animals found/total number of animal)	Loss of Parietal Cells observed
WT (C57BL/6)	0% (0/20)	0% (0/20)
IL-17RA−/−	37% (7/19)***	26% (5/19)***
IL-17RB−/−	4% (1/23)	0% (0/23)

*** p<0.001.

### IL-17RB is required for Th2-mediated response during *H. pylori* infection

**Figure 4:**
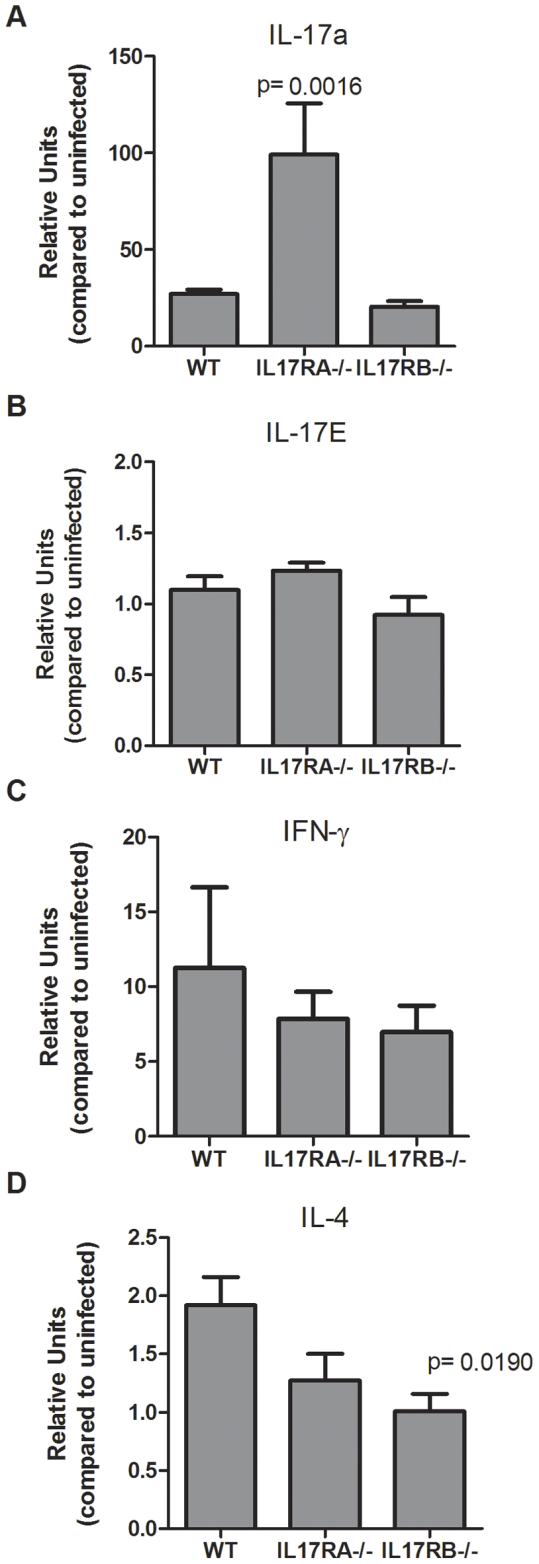
T helper cell cytokine expression is affected by IL-17R deficiencies. Real time rtPCR was performed on stomach tissue of *H. pylori* (strain SS1)- infected mice. Relative units of IL-17A (A), IL-17E (B), IFNγ (C) and IL-4 (D) were measured at 3 months post-infection. Relative units also known as normalized expression are a measure of the relative expression calibrated to expression in uninfected wild-type mice using GAPDH as the endogenous control. Each time point represents 5–6 mice per group.

*H. pylori* infection is known to elicit a mixed Th1, Th2, and Th17 response in the gastric mucosa [1], [5]. When IL-17RA−/− mice were infected with *H. pylori*, we observed a striking, significant increase in the expression of IL-17A [17]. Published studies demonstrate this increase in IL-17A in the IL-17RA−/− mice is the result of the loss of a negative feedback loop [41]. Therefore, in this study, we investigated not just the level of Th17 cytokine, IL-17A, but also other T helper cytokines in the IL-17RA−/−, IL-17RB−/− and WT mice. As expected the *H. pylori-* infected IL-17RA−/− mice had significantly higher expression of IL-17A (Figure 4A). As a control we investigated whether a IL-17E was also involved in a negative feedback loop. Our gene transcription data indicates there is no negative feedback loop involved in expression of IL-17E in the *H. pylori-* infected IL-17RB−/− mice (Figure 4B). IL-17RB−/− mice expressed similar levels of the IL-17E cytokine as both IL-17RA−/− and WT mice.

T cell responses are necessary for *Helicobacter* infection to result in a gastritis response [6], [42], [43]. Moreover, inflammatory cytokine levels often correlate with the levels of inflammation. Therefore, we investigated the expression of T helper cytokine expression in *H. pylori-* infected mice. While IL-17A and IFNγ were induced in the IL-17RB−/− and the WT mice, there was no significant difference in the levels of expression of these cytokines (Figure 4C). This is not surprising since there was no significant difference in the infiltration of immune cells into the stomach after infection. Interestingly, the IL-17RB−/− mice do exhibit significantly reduced expression of IL-4, a marker for Th2 responses (Figure 4D). While Th2 cytokines have been detected in *H. pylori* infection [44]–[48], the role of Th2 cells and the B cell response is not well understood.

### Loss of IL-17RB signaling results in reduced serum antibody

**Figure 5:**
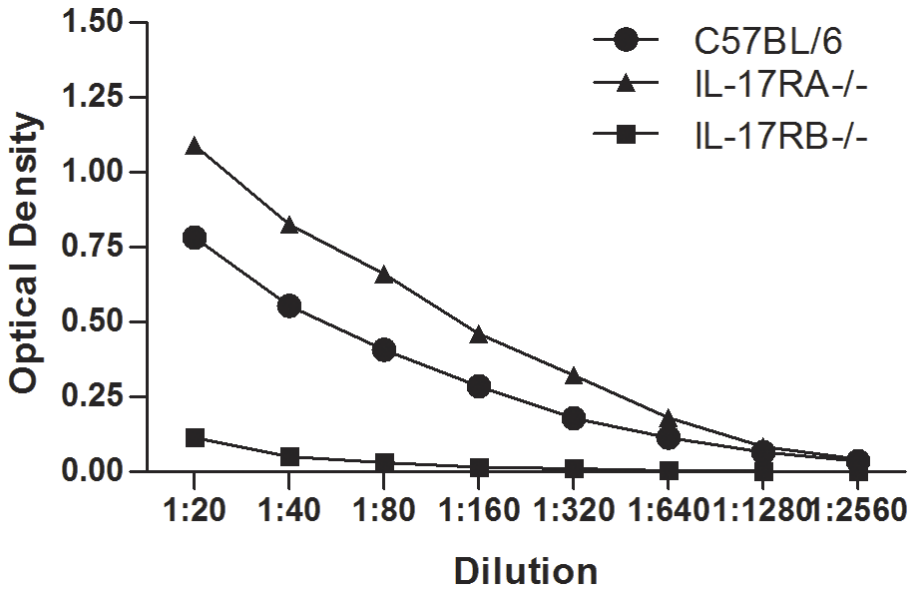
*H. pylori*-specific IgG1is reduced in IL-17RB deficient mice during *H. pylori* infection. Isotype antibodies specific for *H. pylori* (strain SS1) were quantitated by enzyme-linked immunosorbent assay (ELISA). Levels of *H. pylori*- specific IgG1 in the serum from SS1 infected mice were measured at 3 months post infection. 9–10 mice (independent serum samples) were used for each mouse group.

To determine whether reduced levels of IL-4 in the *H. pylori-* infected IL-17RB−/− mice compared to WT or IL-17RA−/− mice results in a change in Th2 responses, we investigated the *H. pylori-*specific antibody responses in the serum. *H. pylori-*specific ELISAs were performed to quantify IgA, a mucosal antibody, IgG1, a Th2 antibody, and IgG2a, a Th1 antibody. As previously reported, IL-17RA−/− mice have elevated *H. pylori-*specific antibody titers in their serum (Figure 5, Table 2, Table 3, and Table 4). Interestingly, while there was more IgA and IgG2a in the *H. pylori-* infected WT mice compared to the *H. pylori-*infected IL-17RB−/− mice, the most significant difference was in levels of IgG1 (Figure 5, Table 4). *H. pylori-*specific IgG1 was only detected in 60% of *H. pylori-*infected IL-17RB−/− mice with the average antibody titer of less than 20. In contrast, *H. pylori-*specific IgG1 was detected in 100% of *H. pylori-*infected WT mice with the average antibody titer of greater than 320. These data suggest that the reduced expression of IL-4 in the *H. pylori-* infected IL-17RB−/− mice do translate to a reduced ability to stimulate an IgG1 response in the serum against *H. pylori*. One previous study reported that µMT (−/−) mice (B lymphocyte-deficient) harbored decreased numbers of *H. pylori* compared to WT mice, and suggested that the humoral immune response might enhance *H. pylori* colonization of mice [49]. In this study an altered IgG1 response does not alter *H. pylori* colonization of the mice. Our data supports the previous findings that endogenous IL-4 levels may not be a major contributor to control of *H. pylori* [50]. Moreover, in murine studies, T cell immunity rather than humoral immunity appears to be required for protection [51], [52].

**Table 2:** *H. pylori-* specific IgA titers in the serum of *H. pylori* (strain SS1)-infected mice infected for 3 months.

IgA	WT	IL-17RA−/−	IL-17RB−/−
1	20	160	BD
2	BD	160	BD
3	BD	1280	BD
4	BD	1280	BD
5	BD	1280	BD
6	80	640	BD
7	BD	80	BD
8	20	1280	160
9	BD	640	20
10	20		BD
**Average**	**12.7**	**756**	**18**

(BD, below detection).

**Table 3:** *H. pylori-* specific IgG2a titers in the serum of *H. pylori* (strain SS1)-infected mice infected for 3 months.

IgG2a	WT	IL-17RA−/−	IL-17RB−/−
1	20	640	20
2	20	640	20
3	20	1280	20
4	20	>2560	BD
5	40	640	20
6	20	1280	BD
7	40	640	BD
8	320	640	40
9	BD	640	BD
10	20		BD
**Average**	**55.6**	**995.6**	**13.3**

(BD, below detection).

**Table 4:** *H. pylori-* specific IgG1 titers in the serum of *H. pylori* (strain SS1)-infected mice infected for 3 months.

IgG1	WT	IL-17RA−/−	IL-17RB−/−
1	640	320	BD
2	40	320	40
3	20	320	20
4	40	640	20
5	20	>2560	BD
6	80	640	40
7	320	320	BD
8	>2560	1280	20
9	160	640	20
10	1280		BD
**Average**	**516**	**782.2**	**16**

(BD, below detection).

In summary, *H. pylori-*infected IL-17RB−/− mice do not exhibit the increased B cell infiltrate and increased inflammation observed in the *H. pylori-*infected IL-17RA−/− mice. Rather, *H. pylori* infection of IL-17RB−/− mice results in similar levels of colonization, inflammation and Th1 and Th17 cytokines compared to *H. pylori-* infected WT mice. *H. pylori*- infected IL-17RB−/− mice did express lower levels of IL-4 in their gastric mucosa and had reduced or undetectable levels of *H. pylori-*specific IgG1 compared to infected WT mice. These data suggest that reduced Th2 responses do not change the course of infection, and *H. pylori* colonization and levels of gastritis are similar in the IL-17RB−/− mice compared to WT mice.

## References

[1] Algood HM, Cover TL (2006) *Helicobacter pylori* persistence: an overview of interactions between *H. pylori* and host immune defenses. Clin Microbiol Rev 19: 597–613. 17041136 10.1128/CMR.00006-06 PMC1592695

[2] Correa P, Houghton J (2007) Carcinogenesis of *Helicobacter pylori* . Gastroenterology 133: 659–672. 17681184 10.1053/j.gastro.2007.06.026

[3] Blaser MJ, Atherton JC (2004) *Helicobacter pylori* persistence: biology and disease. J Clin Invest 113: 321–333. 14755326 10.1172/JCI20925 PMC324548

[4] Dixon MF, Genta RM, Yardley JH, Correa P (1996) Classification and grading of gastritis. The updated Sydney System. International Workshop on the Histopathology of Gastritis, Houston 1994. Am J Surg Pathol 20: 1161–1181. 8827022 10.1097/00000478-199610000-00001

[5] Wilson KT, Crabtree JE (2007) Immunology of *Helicobacter pylori*: insights into the failure of the immune response and perspectives on vaccine studies. Gastroenterology 133: 288–308. 17631150 10.1053/j.gastro.2007.05.008

[6] Eaton KA, Mefford M, Thevenot T (2001) The role of T cell subsets and cytokines in the pathogenesis of *Helicobacter pylori* gastritis in mice. Journal of Immunology 166: 7456–7461. 10.4049/jimmunol.166.12.7456 11390498

[7] Kandulski A, Wex T, Kuester D, Peitz U, Gebert I, et al (2008) Naturally occurring regulatory T cells (CD4+, CD25high, FOXP3+) in the antrum and cardia are associated with higher *H. pylori* colonization and increased gene expression of TGF-beta1. Helicobacter 13: 295–303. 18665940 10.1111/j.1523-5378.2008.00612.x

[8] Harris PR, Wright SW, Serrano C, Riera F, Duarte I, et al (2008) Helicobacter pylori gastritis in children is associated with a regulatory T-cell response. Gastroenterology 134: 491–499. 18242215 10.1053/j.gastro.2007.11.006

[9] Serrano C, Wright SW, Bimczok D, Shaffer CL, Cover TL, et al. (2013) Downregulated Th17 responses are associated with reduced gastritis in Helicobacter pylori-infected children. Mucosal Immunol. 10.1038/mi.2012.133 PMC3775337 23299619

[10] Rad R, Brenner L, Bauer S, Schwendy S, Layland L, et al (2006) CD25+/Foxp3+ T cells regulate gastric inflammation and Helicobacter pylori colonization in vivo. Gastroenterology 131: 525–537. 16890606 10.1053/j.gastro.2006.05.001

[11] Raghavan S, Suri-Payer E, Holmgren J (2004) Antigen-specific in vitro suppression of murine Helicobacter pylori-reactive immunopathological T cells by CD4CD25 regulatory T cells. Scand J Immunol 60: 82–88. 15238076 10.1111/j.0300-9475.2004.01447.x

[12] Raghavan S, Fredriksson M, Svennerholm AM, Holmgren J, Suri-Payer E (2003) Absence of CD4+CD25+ regulatory T cells is associated with a loss of regulation leading to increased pathology in Helicobacter pylori-infected mice. Clin Exp Immunol 132: 393–400. 12780684 10.1046/j.1365-2249.2003.02177.x PMC1808721

[13] Raghavan S, Quiding-Jarbrink M (2012) Immune modulation by regulatory T cells in *Helicobacter pylori*-associated diseases. Endocr Metab Immune Disord Drug Targets 12: 71–85. 22214337 10.2174/187153012799278974

[14] Mizuno T, Ando T, Nobata K, Tsuzuki T, Maeda O, et al (2005) Interleukin-17 levels in *Helicobacter pylori-*infected gastric mucosa and pathologic sequelae of colonization. World J Gastroenterol 11: 6305–6311. 16419159 10.3748/wjg.v11.i40.6305 PMC4320334

[15] Caruso R, Fina D, Paoluzi OA, Del Vecchio Blanco G, Stolfi C, et al (2008) IL-23-mediated regulation of IL-17 production in *Helicobacter pylori-*infected gastric mucosa. Eur J Immunol 38: 470–478. 18200634 10.1002/eji.200737635

[16] Delyria ES, Redline RW, Blanchard TG (2008) Vaccination of Mice Against *H. pylori* Induces a Strong Th-17 Response and Immunity That Is Neutrophil Dependent. Gastroenterology. 10.1053/j.gastro.2008.09.017 PMC4960660 18948106

[17] Algood HM, Allen SS, Washington MK, Peek RM Jr, Miller GG, et al (2009) Regulation of gastric B cell recruitment is dependent on IL-17 receptor A signaling in a model of chronic bacterial infection. J Immunol 183: 5837–5846. 19812196 10.4049/jimmunol.0901206 PMC2834183

[18] Algood HM, Gallo-Romero J, Wilson KT, Peek RM Jr, Cover TL (2007) Host response to *Helicobacter pylori* infection before initiation of the adaptive immune response. FEMS Immunol Med Microbiol 51: 577–586. 17919297 10.1111/j.1574-695X.2007.00338.x

[19] Kabir S (2011) The role of interleukin-17 in the *Helicobacter pylori* induced infection and immunity. Helicobacter 16: 1–8. 10.1111/j.1523-5378.2010.00812.x 21241406

[20] Gaffen SL (2009) Structure and signalling in the IL-17 receptor family. Nat Rev Immunol 9: 556–567. 19575028 10.1038/nri2586 PMC2821718

[21] Kramer JM, Yi L, Shen F, Maitra A, Jiao X, et al (2006) Evidence for ligand-independent multimerization of the IL-17 receptor. J Immunol 176: 711–715. 16393951 10.4049/jimmunol.176.2.711 PMC2973994

[22] Ho AW, Gaffen SL (2010) IL-17RC: a partner in IL-17 signaling and beyond. Semin Immunopathol 32: 33–42. 20012905 10.1007/s00281-009-0185-0 PMC2837117

[23] Rickel EA, Siegel LA, Yoon BR, Rottman JB, Kugler DG, et al (2008) Identification of functional roles for both IL-17RB and IL-17RA in mediating IL-25-induced activities. J Immunol 181: 4299–4310. 18768888 10.4049/jimmunol.181.6.4299

[24] Iwakura Y, Ishigame H, Saijo S, Nakae S (2011) Functional specialization of interleukin-17 family members. Immunity 34: 149–162. 21349428 10.1016/j.immuni.2011.02.012

[25] Ramirez-Carrozzi V, Sambandam A, Luis E, Lin Z, Jeet S, et al (2011) IL-17C regulates the innate immune function of epithelial cells in an autocrine manner. Nat Immunol 12: 1159–1166. 21993848 10.1038/ni.2156

[26] Kolls JK, Linden A (2004) Interleukin-17 family members and inflammation. Immunity 21: 467–476. 15485625 10.1016/j.immuni.2004.08.018

[27] DeLyria ES, Nedrud JG, Ernst PB, Alam MS, Redline RW, et al (2011) Vaccine-induced immunity against Helicobacter pylori in the absence of IL-17A. Helicobacter 16: 169–178. 21585602 10.1111/j.1523-5378.2011.00839.x PMC3107727

[28] Otani K, Watanabe T, Tanigawa T, Okazaki H, Yamagami H, et al (2009) Anti-inflammatory effects of IL-17A on *Helicobacter pylori*-induced gastritis. Biochem Biophys Res Commun 382: 252–258. 19249291 10.1016/j.bbrc.2009.02.107

[29] Shiomi S, Toriie A, Imamura S, Konishi H, Mitsufuji S, et al (2008) IL-17 is Involved in *Helicobacter pylori-*Induced Gastric Inflammatory Responses in a Mouse Model. Helicobacter 13: 518–524. 19166417 10.1111/j.1523-5378.2008.00629.x PMC2631559

[30] Velin D, Favre L, Bernasconi E, Bachmann D, Pythoud C, et al (2009) Interleukin-17 is a critical mediator of vaccine-induced reduction of *Helicobacter* infection in the mouse model. Gastroenterology 136: 2237–2246 e2231. 10.1053/j.gastro.2009.02.077 19272385

[31] Fort MM, Cheung J, Yen D, Li J, Zurawski SM, et al (2001) IL-25 induces IL-4, IL-5, and IL-13 and Th2-associated pathologies in vivo. Immunity 15: 985–995. 11754819 10.1016/s1074-7613(01)00243-6

[32] Hurst SD, Muchamuel T, Gorman DM, Gilbert JM, Clifford T, et al (2002) New IL-17 family members promote Th1 or Th2 responses in the lung: in vivo function of the novel cytokine IL-25. J Immunol 169: 443–453. 12077275 10.4049/jimmunol.169.1.443

[33] Ye P, Rodriguez FH, Kanaly S, Stocking KL, Schurr J, et al (2001) Requirement of interleukin 17 receptor signaling for lung CXC chemokine and granulocyte colony-stimulating factor expression, neutrophil recruitment, and host defense. J Exp Med 194: 519–527. 11514607 10.1084/jem.194.4.519 PMC2193502

[34] Barman M, Unold D, Shifley K, Amir E, Hung K, et al (2008) Enteric salmonellosis disrupts the microbial ecology of the murine gastrointestinal tract. Infect Immun 76: 907–915. 18160481 10.1128/IAI.01432-07 PMC2258829

[35] Lee A, O'Rourke J, De Ungria MC, Robertson B, Daskalopoulos G, et al (1997) A standardized mouse model of *Helicobacter pylori* infection: introducing the Sydney strain. Gastroenterology 112: 1386–1397. 9098027 10.1016/s0016-5085(97)70155-0

[36] Thompson LJ, Danon SJ, Wilson JE, O'Rourke JL, Salama NR, et al (2004) Chronic *Helicobacter pylori infection* with Sydney strain 1 and a newly identified mouse-adapted strain (Sydney strain 2000) in C57BL/6 and BALB/c mice. Infect Immun 72: 4668–4679. 15271928 10.1128/IAI.72.8.4668-4679.2004 PMC470698

[37] Horvath DJ Jr, Washington MK, Cope VA, Algood HM (2012) IL-23 contributes to control of chronic *Helicobacter pylori* infection and the development of T helper responses in a mouse model. Front Immunol 3: 56. 22566937 10.3389/fimmu.2012.00056 PMC3342083

[38] Giulietti A, Overbergh L, Valckx D, Decallonne B, Bouillon R, et al (2001) An overview of real-time quantitative PCR: applications to quantify cytokine gene expression. Methods 25: 386–401. 11846608 10.1006/meth.2001.1261

[39] Arnold IC, Lee JY, Amieva MR, Roers A, Flavell RA, et al (2011) Tolerance rather than immunity protects from *Helicobacter pylori-*induced gastric preneoplasia. Gastroenterology 140: 199–209. 20600031 10.1053/j.gastro.2010.06.047 PMC3380634

[40] Tegtmeyer N, Wessler S, Backert S (2011) Role of the cag-pathogenicity island encoded type IV secretion system in Helicobacter pylori pathogenesis. FEBS J 278: 1190–1202. 21352489 10.1111/j.1742-4658.2011.08035.x PMC3070773

[41] Smith E, Stark MA, Zarbock A, Burcin TL, Bruce AC, et al (2008) IL-17A inhibits the expansion of IL-17A-producing T cells in mice through “short-loop” inhibition via IL-17 receptor. J Immunol 181: 1357–1364. 18606690 10.4049/jimmunol.181.2.1357 PMC2586908

[42] Eaton KA, Ringler SR, Danon SJ (1999) Murine splenocytes induce severe gastritis and delayed-type hypersensitivity and suppress bacterial colonization in *Helicobacter pylori*-infected SCID mice. Infect Immun 67: 4594–4602. 10456905 10.1128/iai.67.9.4594-4602.1999 PMC96783

[43] Roth KA, Kapadia SB, Martin SM, Lorenz RG (1999) Cellular immune responses are essential for the development of Helicobacter felis-associated gastric pathology. J Immunol 163: 1490–1497. 10415051

[44] Smythies LE, Waites KB, Lindsey JR, Harris PR, Ghiara P, et al (2000) *Helicobacter pylori-*induced mucosal inflammation is Th1 mediated and exacerbated in IL-4, but not IFN-gamma, gene-deficient mice. Journal of Immunology 165: 1022–1029. 10.4049/jimmunol.165.2.1022 10878379

[45] Orsini B, Vivas JR, Ottanelli B, Amedei A, Surrenti E, et al (2007) Human gastric epithelium produces IL-4 and IL-4delta2 isoform only upon *Helicobacter pylori* infection. Int J Immunopathol Pharmacol 20: 809–818. 18179754 10.1177/039463200702000417

[46] Marotti B, Rocco A, De Colibus P, Compare D, de Nucci G, et al (2008) Interleukin-13 mucosal production in *Helicobacter pylori*-related gastric diseases. Dig Liver Dis 40: 240–247. 18243827 10.1016/j.dld.2007.11.021

[47] Hosseini ME, Oghalaie A, Habibi G, Nahvijoo A, Hosseini ZM, et al (2010) Molecular detection of host cytokine expression in *Helicobacter pylori* infected patients via semi-quantitative RT-PCR. Indian J Med Microbiol 28: 40–44. 20061762 10.4103/0255-0857.58727

[48] Kido M, Tanaka J, Aoki N, Iwamoto S, Nishiura H, et al (2010) *Helicobacter pylori* promotes the production of thymic stromal lymphopoietin by gastric epithelial cells and induces dendritic cell-mediated inflammatory Th2 responses. Infect Immun 78: 108–114. 19841072 10.1128/IAI.00762-09 PMC2798197

[49] Akhiani AA, Schon K, Lycke N (2004) Vaccine-induced immunity against *Helicobacter pylori* infection is impaired in IL-18-deficient mice. J Immunol 173: 3348–3356. 15322198 10.4049/jimmunol.173.5.3348

[50] Chen W, Shu D, Chadwick VS (1999) *Helicobacter pylori* infection in interleukin-4-deficient and transgenic mice. Scand J Gastroenterol 34: 987–992. 10563668 10.1080/003655299750025084

[51] Ermak TH, Giannasca PJ, Nichols R, Myers GA, Nedrud J, et al (1998) Immunization of mice with urease vaccine affords protection against *Helicobacter pylori* infection in the absence of antibodies and is mediated by MHC class II-restricted responses. J Exp Med 188: 2277–2288. 9858514 10.1084/jem.188.12.2277 PMC2212427

[52] Pappo J, Torrey D, Castriotta L, Savinainen A, Kabok Z, et al (1999) *Helicobacter pylori* infection in immunized mice lacking major histocompatibility complex class I and class II functions. Infection & Immunity 67: 337–341. 9864234 10.1128/iai.67.1.337-341.1999 PMC96315

